# A Comprehensive Review of the Generalized Anxiety Disorder

**DOI:** 10.7759/cureus.46115

**Published:** 2023-09-28

**Authors:** Aneesh K Mishra, Anuj R Varma

**Affiliations:** 1 Medicine, Jawaharlal Nehru Medical College, Datta Meghe Institute of Higher Education and Research, Wardha, IND; 2 Internal Medicine, Jawaharlal Nehru Medical College, Datta Meghe Institute of Higher Education and Research, Wardha, IND

**Keywords:** cognitive behavioral therapy, inhibitory neurotransmitters, psychotherapy, relaxation methods, generalized anxiety disorder

## Abstract

Excessive, uncontrollable, and usually unjustified worry about certain things is a sign of the mental and behavioral disease known as generalized anxiety disorder (GAD). Genetic research suggests that numerous genes are likely implicated in the development of GAD, even if much is yet unclear about this. As a result, if someone in a family has GAD, there is a high likelihood that someone else will also suffer from the illness, as well as another anxiety disorder. Individuals with GAD are frequently overly bothered about workaday affairs like health, assets, demise, family, accord issues, or effort challenges. Worry frequently interferes with daily functioning. Excessive concern, restlessness, difficulty sleeping, tiredness, irritability, sweating, and trembling are a few symptoms that may be present. For a formal diagnosis of GAD, symptoms must be persistent for at least six months and consistent. Conversion in the amygdala's utilitarian congruence and how it processes fear and anxiety have been linked to generalized anxiety disorder. Neurotransmitters, and particularly the gamma-aminobutyric acid (GABA) variant, have long been known to cause GAD through dysregulating amygdala activity in the brain. Anxiety, concern, or physical symptoms must significantly hinder social, academic, or occupational functioning in order to qualify for a GAD diagnosis. The Diagnostic and Statistical Manual of Mental Disorders-V (DSM-V) provides explicit ethos to aid doctors in identifying this disorder. Psychological therapy based on cognitive behavioral therapy (CBT) principles is effective in reducing anxiety symptoms for short-term treatment of GAD. In this, the patient's thinking ability and methods are focused. The main principle behind CBT is that your thought patterns affect your feelings, which in turn can affect your behavior. Drugs like antidepressants, buspirone, benzodiazepines, and can all be worn to goody GAD. Outside of therapy, patients with anxiety can learn to manage it by practicing relaxation methods, reframing unfavorable ideas, and adopting stress-relieving adjustments. Being socially active and setting aside time for proper self-care are crucial components of managing generalized anxiety disorder.

## Introduction and background

Usually, a person who perceives sadness or difficulties quickly overcomes it, however, in generalized anxiety disorder (GAD), their sadness or anxiety spirals out of control. He cannot regulate his stress, and his regular life begins to be affected by his fear of the unexpected. It can occasionally last for a very long time, and this condition affects twice as many women as it does men [1]. In contrast to the projected current prevalence of between 2% and 3%, the expected lifetime prevalence of the Diagnostic and Statistical Manual of Mental Disorders-IV (DSM-IV) GAD in the United States is about 5%. Gender, race, and social class all have different rates of illness. GAD affects 6.8 million adults or 3.1% of the US population, yet only 43.2% are receiving treatment. This estimated increase in anxiety disorders in the United States is believed to be due to excessive use of social media, lack of proper sleep, genetic factors, and environmental factors [2]. Few thorough demographic modules have been the antecedent brainy disease or brainy healthcare code and fulfillment, the 2016 Indian National Mental Health Survey (NMHS) steered to improve brainy health throughout the nation [3]. While generalized anxiety disorder is frequently observed in general practice, the more dramatic varieties of anxiety disorders, such as psychiatric illness and fetishes, have received more detailed research [4].

Types of generalized anxiety disorders

Social Phobia

An extreme awe of one or more cordial or consummation bearings that last for an extended period is the definition of social anxiety, a common disorder. Evident reluctance is one of the earliest indications of collective disquiet, which may grow into a specific personality type (reduced agreeableness and elevated psychoticism) and the emergence of adverse cognitive biases [5].

Obsessive-Compulsive Disorder (OCD)

It includes obsessions, compulsions, or frequently both. It is the fourth most commonplace brainy anarchy after social phobia, alcoholism, and depression, with a lifetime prevalence in population surveys of 1.6%. OCD severity varies significantly from person to person. Even from their own family, people can frequently conceal their OCD, although it can interfere with relationships and make it difficult to study or work [6].

Post-traumatic Stress Disorder (PTSD)

Due to its prevalence, long-term nature, and impact on daily functioning, it is a notable public health matter. Military psychiatrists and early psychoanalysts have understood and studied PTSD since antiquity. Still, today, more than ever, it is a hot topic due to the numerous events like terrorist attacks or weather disasters recently in France and elsewhere. Reexperiencing, avoidance, hyperarousal, and changes in cognition and mood are the four essential characteristics of this illness [7].

Panic Disorder

The hallmarks of anxiety disorders such as panic disorder are brief, intense moments of dread accompanied by physical symptoms, including breathing difficulties, chest pain, heart palpitations, nausea, and stomach aches [8]. Certain generalizations regarding the illnesses have been made, including abnormalities in the limbic system, anomalies in the hypothalamic-pituitary-adrenal axis, genetic makeup, additional humdrum peril ingredient for dread anarchy encompass womanish and familial antiquity of dread, and some form of autism [9].

## Review

Methodology

**Figure 1:**
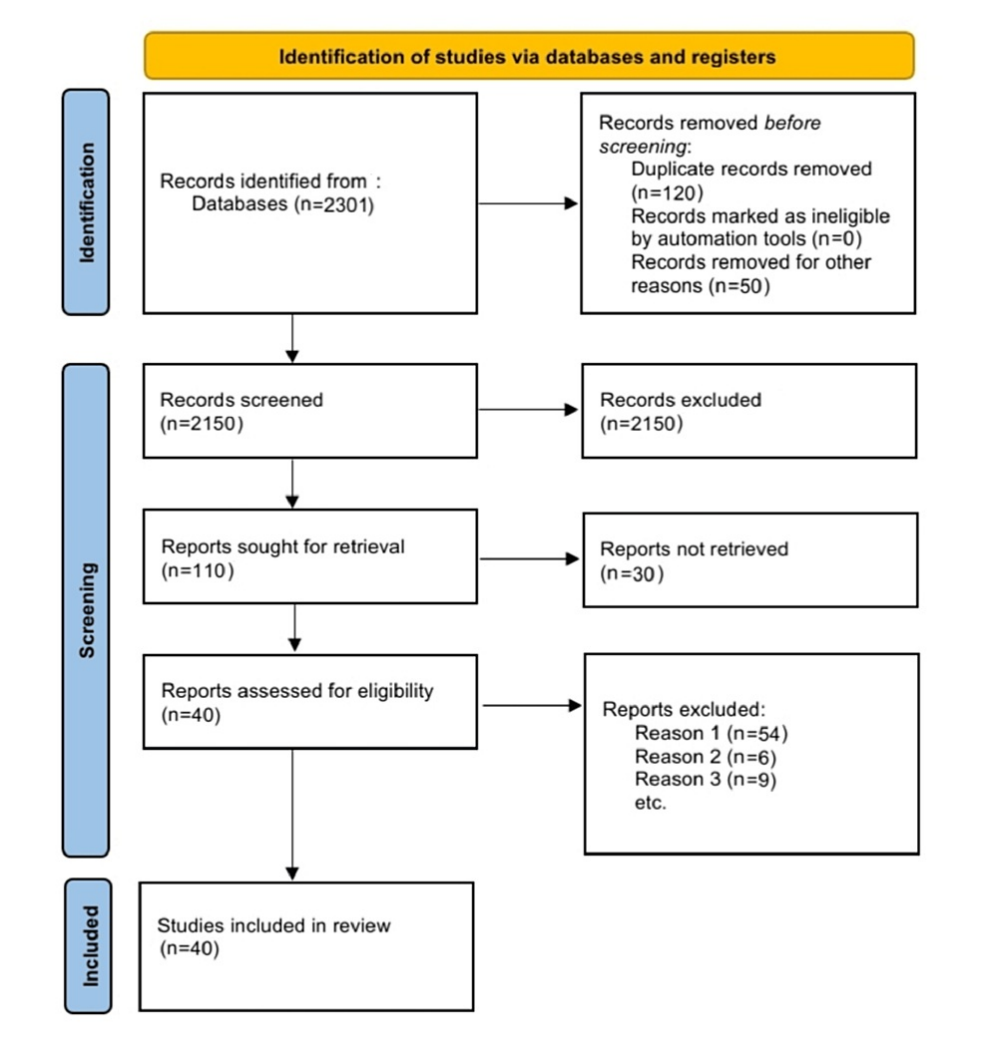
The selection process of articles used in this study. PRISMA: Preferred Reporting Items for Systematic Reviews and Meta-Analyses

The terms "generalized anxiety," "panic disorder," "social phobia," and "neuroticism" were searched in a database like "PubMed." There were only English-related results shown. The most recent report from a similar study was utilized if there were multiple published reports. We only considered reviews that also included original data. The Preferred Reporting Items for Systematic Reviews and Meta-Analysis (PRISMA) flow diagram for the search is shown in Figure 1.

Pathophysiology

A lively and active field, research into the pathophysiology of GAD typically involves the intersection of genetics and brain structure. A connection has been made between generalized anxiety disorder and a shift in the handy congruence of the amygdala and how it processes despair and misery. The etiology of generalized anxiety disorders is complicated by so many ingredients, including genetics, an imbalance of levels of neurotransmitters, and others.

Genetics

Genetic factors predispose certain people to develop a generalized anxiety disorder. Depression is referred to as genetic depression because it affects about 40% of depressed individuals. The chance of acquiring depression can increase up to three times if a close relative, such as a parent or sibling, is affected by the illness. Genes may raise the likelihood of inheriting depression, but other environmental variables ultimately cause the condition. The serotonin transporter gene has also been linked to genetic depression, according to research [10].

Noradrenergic Activity

Patients with GAD had higher levels of plasma norepinephrine (NE) and free 3-methoxy-4-hydroxyphenethylene glycol (MHPG) and lower levels of alpha 2-adrenoreceptor. Patients with GAD appear to have enhanced noradrenergic activity. Higher catecholamine levels in GAD patients could result in a down-regulation of presynaptic alpha 2-adrenoreceptor [11].

Role of Gamma-Aminobutyric Acid (GABA)

It is an inhibitory neurotransmitter identified in numerous studies to be dysregulated, particularly the GABA variation, which has long been found to increase amygdala activity in the brain and result in GAD. After a neurotransmitter release brought on by stress, the body finds it difficult to relax when the level of GABA is deficient. Depression, sleeplessness, and mood disorders are caused by this [12].

Role of Serotonin Receptor in Generalized Anxiety Disorder

Multiple receptors are activated by serotonin, which controls a variety of physiological processes. Abnormalities in these receptor systems have been linked to several brain problems, like dreed, blahs, lunacy, migraine, sleep, cognition, and feeding. While less abundant in the primary sensory regions, the 5-hydroxytryptamine (5-HT1A) receptor is expressed in high concentrations in the limbic, temporal, and prefrontal cortex [13].

Role of Dopamine in Generalized Anxiety Disorder

Numerous brain regions may be affected by dopamine's effect on anxiety modulation. According to some studies, the dopaminergic systems in the mesolimbic, mesocortical, and nigrostriatal regions affect anxiety. The dopamine D1 and D2 receptor loops and misery are involved [14].

Risk factors

Use of tobacco, alcohol, and cannabis, not to mention avoidance, adverse assessments of brio drill, and occupational characteristics were all found as risk factors. The risk factor that has been investigated the most is cigarette smoking. Evidence supports tobacco as a problematic element for frenzy and agoraphobia [15].

Characteristics

GAD people contain a broad range of features and varying degrees of severity. Some patients can place more emphasis on a particular symptom than others that are often related to GAD's highly enhanced physiological arousal, emotional lability and muscular stress, fatigue, restlessness, and difficulty sleeping. Some other symptoms seen in a person suffering from GAD include an excessive attachment to any unnecessary thing, unnecessary emphasis on something, lack of concentration, weakening of memory, rapid heartbeat, and inability to remain stable and calm. Also, symptoms like physical weakness, irritability, restlessness, etc. are included [16].

Specifications for diagnosing

Several different scales are used to determine a diagnosis and evaluate severity. A rate of 10 or higher has appropriate indicative subtlety and idiosyncrasy and the GAD-7 has been approved as an indicative gizmo and an austerity-grade extent. There is a correlation between higher GAD-7 scores and spare serviceable ruination. Even though the scope was created and ratified using DSM-IV decency, it is still clinically fruitful due to modest modifications in the GAD diagnostic criteria. Its trendy indicative ascetic dispatch measure for angst cloaking, scrutiny, and acerbity estimate is the GAD-7 [17].

Generalized Anxiety Disorder Indicative Conventionalities Akin to the Diagnostic and Statistical Manual of Mental Disorders (DSM-IV) Criteria

Its purpose is to provide a structure for organizing each of the mentioned conditions, to create diagnostic standards, and to categorize diseases. In Table 1, the DSM-IV criteria are displayed [18].

**Table 1:** DSM-IV criteria for generalized anxiety disorder. DSM: Diagnostic and Statistical Manual of Mental Disorders

Exorbitant dread also misgiving (≥6 months) concerning variety of occasions or bustle
Controlling worry is difficult
Angst and anguish accompanied by symptoms like restlessness, easily becoming tired, having trouble focusing, or bustling bare, impatience, muscle tightness, and disturbed sleep
The focus of concern and worry extends beyond the signs of Axis I disease

Generalized Anxiety Disorder Convention According to the Diagnostic and Statistical Manual of Mental Disorders (DSM-V)

Diagnostic criteria used to characterize GAD in the final version of the DSM-V were as follows: extreme tension and worry for a minimum of six months around various occasions or enterprises, like consummation in the workplace or school. It is challenging for the person to keep the worry under the curb. Dreed is related to unease or a sense of tension or apprehension, being easily worn out, inability to focus or mental disorientation, irritation, tight muscles, sleep disruption, inability to decline or keep asleep, or restive, uncomfortable sleep [19].

Extensive gloom and generalized anxiety disorders are co-occurring, and this combination often results in worse outcomes than either disorder by itself. Additionally, according to epidemiological data, 59% of people with this disorder meet the requirements for major depressive disorder (MDD). So, the most prevalent type of depression and anxiety comorbidity is MDD and GAD. Even though co-occurring GAD and MDD are more common in some clinical samples than others, this clinical problem is unquestionably present in people undergoing psychiatric care [20].

Pharmacological management

Based on different types of studies, it has been reported that drugs based on serotonin and non-epinephrine are broadly used because of their higher efficacy and rapid improvement of symptoms and relief for the patients. These drugs have a wide range of benefits [21]. To reduce anxiety, one can take support of different types of therapy like cognitive therapy, using depression and anxiety-reducing drugs [22].

Selective serotonin reuptake (SSRI) inhibitor

The SSRIs impede the functions of some transporters and, in some cases, affect a few other reuptake processes. Some levels are up, and some groups are down by this. Synaptic 5-HT concentrations are raised by inhibiting 5-HT reuptake, which also raises extra-synaptic diffusion. SSRI examples are given below.

Fluoxetine

Fluoxetine was created in the early 1970s and was the first SSRI available in the United States in 1987. Fluoxetine improves serotonergic transmission in the brain, but it also purportedly has noradrenergic and dopaminergic actions that contribute to its therapeutic success. It is very effective, which various research proves [23].

Sertraline

Sertraline is an antidepressant. It functions by raising the brain's concentration of the chemical messenger serotonin. It lowers the psychological and physiological signs of depression, as well as the symptoms of anxiety and some other diseases. Sertraline, the finest remedy for reducing treatment disruption, is the second excellent remedy for decisive echo in persons who do not stop their regimen due to adverse fallout. Sertraline appears to be the most reasonably priced drug for treating GAD patients [24].

Paroxetine

Paroxetine is now ratified to cure social anxiety, panic disorder, and depression. Additionally, it is worn to treat persistent headaches and other brain disorders. The strongest inhibitor of serotonin (5-hydroxytryptamine {5-HT}) reuptake among all currently prescribed antidepressants, including the SSRI family, is paroxetine, a phenylpiperidine derivative. It is a much weaker than average NE uptake inhibitor, yet it is still more effective at this location than the other SSRIs [25].

Citalopram

Citalopram hydrobromide was declared the most efficient selective serotonin reuptake inhibitor by the FDA in 1998. It is an edict remedy in the WHO model agenda of essential medications for treating depressive disorders. Serotonergic activity in the central nervous system is potentiated due to its reduction of serotonin absorption by CNS neurons [26].

Selective serotonin-norepinephrine reuptake (SNRI) inhibitor

It conveys messages among brain cells and boosts well-being, an optimistic outlook, appetite, and social behavior while regulating internal body clocks and sleep-wake patterns. Dual serotonin and noradrenaline reuptake inhibition may be advantageous since it addresses a broader range of symptoms than other antidepressants. Some examples are given below.

Venlafaxine

Venlafaxine is a perfect remedy, broadly used to relieve patients. They were first blocking serotonin and then norepinephrine. Medical conditions and treatment responses are considered while determining the dosage [27].

Duloxetine

The use of duloxetine as an antidepressant and other members of the pharmacological group of serotonin-norepinephrine reuptake inhibitors to cure brainy diseases and correct the chemical imbalance in the brain is approved. Its action method depends on the central nervous system's reuptake inhibition of norepinephrine (NE) [28].

Anxiolytics

Antianxiety drugs, also called anxiolytics, are administered to treat and lessen anxiety brought on by various anxiety disorders. These drugs have a quick start of the action and can establish habits. They are typically only prescribed for short-term use as a result. It is not advised for those who have an antiquity of stuff misdeed. Some examples of anxiolytics are given below.

Buspirone

The drug buspirone is a popular anxiolytic; buspirone has become increasingly popular recently. Anxiety disorders are typically treated with buspirone. SSRIs are repeatedly worn as an additional cure for patients who do not react to or cannot tolerate their adverse effects [29].

Benzodiazepines 

Benzodiazepines are helpful for anxiety disorders because they act immediately. However, the effectiveness of benzodiazepines can differ depending on the form of drug illness. The popularity of benzodiazepines has persisted for a number of reasons, including their consistent efficacy in reducing anxiety, tension, and various physical symptoms of anxiety, rapid onset of therapeutic action, relatively good tolerability, ability to be administered on an as-needed basis, and comparatively safe overdose rate. Benzodiazepines are successful in the cure of such diseases [30].

Role of psychotherapy in generalized anxiety disorder

Psychotherapy aims to improve a person's relationships, social skills, and mental and physical health. Additionally, it works to eliminate or diminish negative feelings, thoughts, compulsions, or behaviors. Two regularly employed psychological therapies are supportive, interpersonal, and cognitive behavioral therapy [31].

Cognitive-Behavioral Therapy (CBT)

CBT is regarded as the "gold standard" [32]. Reduced anxiety, more accessible job performance, and an overall higher quality of life are all benefits of this [33]. The typical understanding of CBT is that it is an efficient, dexterity-core remedy to comfort patients to revise their faith and behaviors [34]. CBT is a common strategy for people who need to improve their response to medicine and a successful approach for those who don't respond to medication, want to stop taking their drugs, or don't respond to treatment [35].

Interpersonal Therapy (IPT)

Although there has been far less research on IPT's effectiveness in treating anxiety disorders, it has successfully treated eating and mood disorders. In patients with eating and mood disorders, IPT reliably improved anxiety symptoms [36]. IPT helps patients strengthen social networks and boost the quality of their relationships by emphasizing traumatic events, including grieving, interpersonal conflicts, life changes, or social isolation [37].

Computer-Aided Psychotherapy (CP)

Computer-assisted psychotherapy is very useful for persons with anxiety disorders, requiring less therapist time, expediting access to care, and decreasing travel time. Computers that are standalone or connected to the Internet, handheld devices, interactive voice response phones, DVD players, and cell phones can all be used to offer CP [38].

Supportive Therapy

Active and inactive conditions were separated from it. All psychiatric treatments were compared to waiting lists and standard care, including functional, supportive therapy. Compared with CBT, ST was an additional psychological treatment [39].

Applied Relaxation (AR) Therapy

Well-established and successful treatment for phobias, panic attacks, and, later, generalized anxiety disorder is called applied relaxation therapy (AR). Early therapy sessions assist individuals in identifying their anxiety's warning symptoms. Throughout the week, clients are instructed to pay attention to and note the events that cause them stress and their responses to these situations. During this monitoring process, they can differentiate between cognitive, affective, physiological, and behavioral cues. AR aims to calm down quickly and use this ability when necessary to lessen physiological reactions and, as a result, the anxiety cycle [40].

## Conclusions

Generalized anxiety disorder are a category of mental disorders that distinguish themselves from other problems with two key features fear and worry. Fear is an emotion experienced in response to an impending danger. On the other hand, anxiety is an emotional state experienced in anticipation of a possible future threat. Patients with GAD exhibit a substantial degree of impairment and disability. Anxiety disorders are associated with cognitive impairment such as reduced processing speed, attention, and inhibition, which may be exacerbated by antianxiety medications. People with generalized anxiety disorder worry about entirely unimportant things, distinguishing them from typical worrying. Additionally, they struggle to get through the day because they constantly fret about potential outcomes. Women seem to have this condition on average twice as often as males. Anxiety disorder can also be caused by any disease related to the body, such as thyroid, asthma, sugar, or heart disease. The self-reported questionnaire diagnoses and appraises grievousness and generalized anxiety disorder. DSM-IV and DSM-V criteria are used widely for the diagnosis of this. The most popular treatments are medication and psychotherapy. Several regimens are beneficial in the management of such conditions. The pharmaceutical classes SSRI and SNRI are among the first-line therapies. People with GAD can use various strategies, including meditation and other stress-reduction exercises, including aerobics and soothing exercises. The challenge of GAD is that anxiety is a ubiquitous emotion, and so it can be difficult to know when anxiety has crossed the line of too much, for this reason, it is important to be aware of its symptoms. Seek immediate medical help if any symptoms are felt. Mental illness carries serious consequences throughout society and calls for new strategies for prevention and intervention. To accomplish these strategies, early detection of mental health issues is an essential process. Therefore, there is a need for more research and study on this in the future.
